# Gibberellin-regulated proteins: Emergent allergens

**DOI:** 10.3389/falgy.2022.877553

**Published:** 2022-09-09

**Authors:** T. Iizuka, A. Barre, P. Rougé, D. Charpin, E. Scala, B. Baudin, T. Aizawa, H. Sénéchal, P. Poncet

**Affiliations:** ^1^ Protein Science Laboratory, Hokkaido University, Sapporo, Japan; ^2^ UMR 152 Pharma-Dev, Toulouse 3 University, Toulouse, France; ^3^ APPA, Marseille, France; ^4^ “Clinical and Laboratory Molecular Allergy” Unit, Istituto Dermopatico Dell’Immacolata—IRCCS, Rome, Italy; ^5^ Biochemistry Department, Armand Trousseau Children Hospital, APHP, Paris, France; ^6^ “Allergy / Environment” Research Team, Armand Trousseau Children Hospital, APHP, Paris, France; ^7^ Immunology Department, Institut Pasteur, Paris, France

**Keywords:** gibberellin-regulated protein, pollen food allergy syndrome, food allergy, pollen allergy, 3D structure

## Abstract

About 10 years ago, a protein family was shown for the first time to contain allergenic members, gibberellin-regulated protein (GRP). The first reported member was from peach, Pru p 7. One can hypothesize that it was not detected before because its physicochemical characteristics overlap with those of lipid transfer protein (LTP), a well-known allergen, or because the exposure to GRP increased due to an increase in the gibberellin phythormone level in plant food, either exogenous or endogenous. Like LTPs, GRPs are small cationic proteins with disulfide bridges, are resistant to heat and proteolytic cleavage, and are involved in the defense of the plant. Besides peach, GRP allergens have been described in Japanese apricot (Pru m 7), sweet cherry (Pru av 7), orange (Cit s 7), pomegranate (Pun g 7), bell pepper (Cap a 7), strawberry (Fra a GRP), and also in pollen with a restriction to Cupressaceae tree family (Cup s 7, Cry j 7, and Jun a 7). IgE cross-reactivities were described between GRPs, and the reported peach/cypress and citrus/cypress syndromes may therefore be explained because of these GRP cross-reactivities. GRPs are clinically relevant, and severe adverse reactions may sometimes occur in association with cofactors. More than 60% and up to 95% sequence identities are calculated between various allergenic GRPs, and three-dimensional models show a cleft in the molecule and predict at least three epitopic regions. The structure of the protein and its properties and the matrix effect in the original allergenic source should be unraveled to understand why, despite the ubiquity of the protein family in plants, only a few members are able to sensitize patients.

## Introduction

Since the characterization, in 2013, of Pru p 7 (formerly peamaclein), a member of the gibberellin-regulated protein (GRP) family, only a few other members were characterized as allergens although these plant proteins are ubiquitous and share conserved amino-acid sequences. Therefore, allergenicity parameters of GRPs are intriguing and remain to be deciphered. This perspective/review will discuss different factors such as cross-reactivity, epitope prediction, and ligand/cofactor interactions.

The name of the allergen family GRP appeared for the first time in a publication of Inomata et al. in 2016 that described some clinical symptoms associated to Pru p 7 sensitization (1). Pru p 7 was the first described allergen belonging to GRP (2). The family name “gibberellin-regulated protein,” while now well accepted in the field of allergy, might not be the most appropriate and rather should be snakin. Indeed, gibberellin is a plant hormone that regulates very diverse proteins in plants, nonallergenic and allergenic ones, such as, besides snakin, superoxide dismutase, β-1,3-glucanase, calmodulin, or oleosin (3). The protein family was also named snakin/GASA (Gibberellic Acid Stimulated in *Arabidopsis*) when snakin-like proteins were described in *Arabidopsis thaliana* (4).

GRP will be used for allergen family in the rest of the manuscript and snakin-1 will refer to the nonallergenic potato GRP. The name snakin has been given to the protein from potato because of some similarities with a protein from the snake venom, kirstin, a disintegrin protein with hemotoxic properties (5).

## The plant hormone

**Figure 1:**
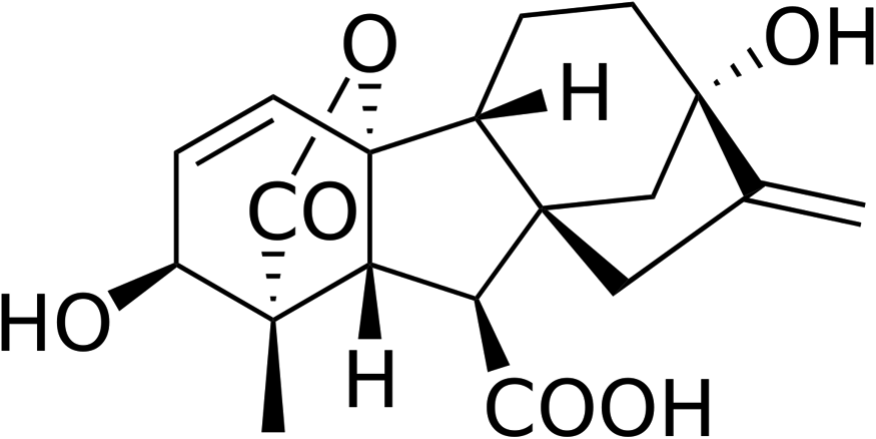
Gibberellin acid (GA_3_), one of the active phythormone in plants. The commercially available GA3 is one of the most used active gibberellin for plant treatment.

Gibberellin (or gibberellic acid: GA) was described for the first time by the phytopathologist Eiichi Kurosawa in 1926 in the Ascomycete parasite of the rice *Gibberella fujikuroi*, which results in a dramatic length increase of the rice stems, defining the so-called foolish seedling rice disease or *Bakanae* (6). Between the years 1935 and 1938, Teijiro Yabuta isolated and purified the hormone in the foolish seedling rice disease (7). Finally, in the years 1954–1955, the tetracyclic diterpenoid chemical structure of GA_1_, GA_2_, and GA_3_ was determined (8) (Figure 1).

Among more than 130 gibberellins identified in plants, fungi, and bacteria, only GA_1_, GA_3_, GA_4_, and GA_7_ are thought to function as bioactive hormones playing a role in plant growth and breaking dormancy (9). Gibberellin and GRP have an important role in plant development, host defense, and redox homeostasis. Furthermore, both biotic (bacteria, viruses, fungi, parasites) and abiotic (drought, temperature, salt content, wounding, flooding) stresses have an impact on endogenous gibberellin and GRP levels (10–13). It was reported, for instance, that a small increase in temperature was able to increase the concentration of endogenous gibberellin (14). Many studies evaluating the impact of environmental modifications on plant hormone, including gibberellins, are published in relation to climate change. Nowadays, synthetic exogenous gibberellins are widely used to increase the yield and/or quality of plant food in modern agriculture (15–17). Several plant foods are submitted to an exogenous gibberellin treatment. These plant foods include grape, cherry, strawberry, pear, tangerine, plum, orange, blueberry, pineapple, tomato, potato, wheat, rice, barley, hop, sunflower, alfalfa (*Medicago*), chili/red pepper, zucchini, salad, spinach, celery, tobacco, or cotton. No data are available on the effect of such treatments on the GRP content and particularly on the snakin protein family, the one shown to contain allergens and, therefore, able to increase the allergenicity of the fruit or vegetable.

## The protein family

GRPs are nonglycosylated, cationic, monomeric proteins with a molecular weight (MW) around 7–8 kDa (63 amino acids) and an isoelectric point (pI) around 9. The protein is hydrosoluble, with hydrophobic patches and a compact very folded globular structure, sometimes leading to overevaluation of its MW in different bio- and physicochemical analytical methods such as analytical ultracentrifugation or unreduced sodium dodecyl sulfate - poly acrylamide gel electrophoresis (SDS-PAGE). GRPs, and more specifically the snakin protein family, belong to the cysteine-rich plant antimicrobial peptide families involved in plant growth and resistance to bacteria, virus, or other microorganisms responsible of plant diseases (18, 19). Twelve cysteines at conserved positions involved in six disulfide bridges confer the protein stability and resistance to heat and proteolysis (2, 20). Because the protein is highly folded, it exhibits conformational IgE epitopes abrogated after reduction of disulfide bonds (20, 21) corresponding to a drastic modification of the original conformation of the protein as shown with Cry j 7 (22). This characteristic may be beneficially used in case of induction of tolerance to this family of allergens. Indeed, since allergen-specific immunotherapy can be hazardous due to difficulties in predicting specific IgE-mediated side effects, reduced form of GRP, devoid of IgE reactivity, could be safely used in specific allergen immunotherapy protocols as previously proposed for pollen and house dust mite allergy as soon as 1998 [reviewed in (23)].

## Snakin-1: the prototypic GRP

**Figure 2:**
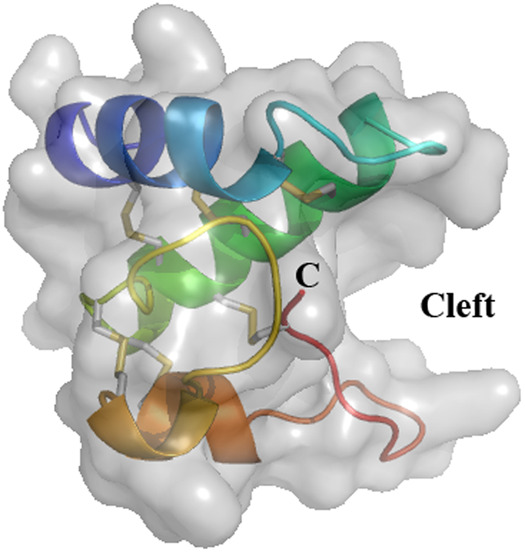
Three-dimensional structure of snakin-1 (PDB 5E5Q). Ribbon representation with surface obtained from crystallography data show three α-helices colored in blue (S2–C13), green (L18–K34), and orange (N43–E46 and C47–K53). The cleft is indicated where a putative ligand could bind. The six disulfide bridges are shown in gray/yellow small bars. C: C-terminal end of the protein. *N*-terminal is masked by the first α-helix (dark blue).

The very first snakin GRP described was the snakin-1 from potato (*Solanum tuberosum*) in 1999. The expression of the snakin-1 gene has been detected in tubers, stems, axillary buds, and young floral buds. Expression levels in petals and carpels from fully developed flowers were much higher than in sepals and stamens (5). Structural and functional (antimicrobial activity) characteristics were thoroughly evaluated (11) including the three dimensional structure by x-ray crystallography (24). The protein is folded in three alpha-helices and exhibits a cleft in which one or more, as yet undetermined, ligands could bind (Figure 2). Allergy to potato is rare and snakin-1 is not yet described as an allergen in potato.

## Plant food GRP allergens

The very first GRP allergen described in 2013 was Pru p 7 in peach (*Prunus persica*) (2). Peach allergic patients selected to unravel Pru p 7 had no IgE against the other allergens from peach and specially no anti-Pru p 3 IgEs. Interestingly Pru p 3, like Pru p 7, has a low MW and a basic pI resulting sometimes in contamination of Pru p 3 purifications with Pru p 7. The characterization of Pru p 7 was refined and confirmed in 2014 (25, 26). Pun g 7 the GRP from pomegranate (*Punica granatum*) was then reported (27) as well as Pru m 7, the GRP from Japanese apricot (*Prunus mume*) (28). Japanese people are exposed to Japanese apricots traditionally consumed marinated in salt and called *umeboshi*. More fruits were suspected to contain allergenic GRP (29–31) and orange (*Citrus sinensis*) Cit s 7 (32) and sweet cherry (*Prunus avium*) Pru av 7 (Inomata N. IUIS/WHO Pru av 7 description. http://wwwallergenorg/searchphp?allergensource=sweet+cherry/searchsource=Search.2019) were characterized. Other citrus species like grapefruit (*Citrus maxima*), tangerine (*Citrus reticulata*), and lemon (*Citrus limon*) contain cross-reactive GRP with orange (33), and a clementine (*Citrus clementina*) GRP is described in the databank Uniprot KB (accession number V4T144) with 100% sequence identity with orange GRP. Very recently, a strawberry GRP, Fra a GRP, was suggested to be sensitizing for one patient also allergic to Japanese cedar pollen, peach, and apple. Symptoms after consumption of strawberries occurred only after physical exercise (34). The sequence is not known and no recombinant was produced.

In contrast to LTP, which is more present in fruit peel than pulp, GRPs are present in peel and in pulp. The amount of Pru p 7, quantified using specific monoclonal antibodies or by qPCR, was reported to be slightly higher in pulp than in peel, higher in ripe fruit, and to vary depending on the cultivar (35, 36). These quantitative variations were also reported for Pun g 7, the pomegranate GRP (27).

Clinical relevance of plant food GRP was assessed using basophil activation test performed with pure GRP either natural or recombinant and also using skin prick test. This was clearly established for Pru p 7, Cit s 7, Pru m 7, Fra a GRP, and Pru av 7 (2, 26, 34, 37) and (Inomata N. IUIS/WHO Pru av 7 description. http://wwwallergenorg/searchphp?allergensource=sweet+cherry/searchsource=Search.2019). Anaphylaxis is often associated with GRP allergies with clinical symptoms such as face and eyelid edemas, or generalized urticaria (1, 38). Severe adverse reactions to GRPs may sometimes occur when cofactors, such as physical exercise, nonsteroidal anti-inflammatory drugs (NSAID), alcohol, proton pump inhibitors, stress, concomitant infections, or menstruations are associated, similarly to other PFAS (32, 38–43).

**Figure 3:**
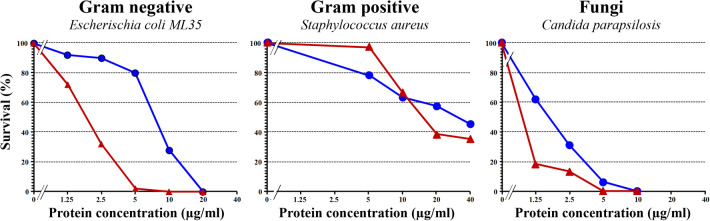
Antimicrobial activity of recombinant Pru p 7 (blue circles) in comparison with snakin-2 (red triangles), a cysteine-rich protein from potato reported to exhibit antimicrobial properties and thus considered as a positive control in the experiment. Various concentrations of recombinant Pru p 7 and snakin-2 were mixed with cell suspension in potassium phosphate buffer (10 mM, pH 6.0) and incubated at 37°C for bacteria (*E. coli* and *S. aureus*) and 30°C for fungi (*C. parapsilosis*) for 1 h. After incubation, the reaction mixture was diluted and plated on agar plates. Tryptic soy broth plates were used for bacteria and Sabouraud agar plates for fungi. After incubation, colonies were counted for calculating the survival rates. A representative experiment is shown and each point was performed in simplicate.

GRPs are produced in recombinant form either in *Escherichia coli* or *Pichia pastoris* (44, 45), and because of the six disulfide bonds, the correct folding should be carefully checked at structural level using proper physicochemical methods such as circular dichroism, nuclear magnetic resonance, and mass spectrometry and also at functional level using assays to assess the antimicrobial activity of the protein. Figure 3 depicts the results of antimicrobial activities against bacteria and fungi of a correctly folded recombinant Pru p 7 as compared to the well-known cysteine-rich antimicrobial protein snakin-2 from potato (46). Prototypic Gram-negative and Gram-positive bacteria and the fungi *Candida parapsilosis* are killed by Pru p 7.

## GRP and PFAS

In parallel, in 2018, an allergen from cypress pollen (*Cupressus sempervirens*), formerly known as BP14 since 2010 (20, 47, 48), was shown to belong to the GRP family (21). Only Cupressaceae pollen were shown to express allergenic GRP more abundantly in *C. sempervirens, Juniperus ashei*, *and Cryptomeria japonica* than in *Hesperocyparis arizonica* (formerly *Cupressus arizonica*) (20, 49). A cross-reactivity was demonstrated between BP14 and Pru p 7 and, in consequence, BP14 could be considered as the missing link (50–52) to explain the pollen/food allergy syndrome (PFAS) described in 2006 between peach and cypress pollen (53). Also, the citrus/cypress pollen PFAS (54) was shown to rely on a cross-reactivity between BP14 (future Cup s 7) and a cationic low MW allergen from citrus fruit (33). The cationic LMW allergen was characterized as Cit s 7 (32) and the cross-reactivity between Cup s 7/Cry j 7 and Cit s 7 was confirmed and published by studying Japanese cedar allergic patients (22, 55). The gene coding for BP14 was fully sequenced from common cypress (*C. sempervirens*) strobili by next-generation sequencing and the protein named Cup s 7 [Poncet P, Aizawa T. IUIS/WHO description of Cup s 7, http://wwwallergenorg/searchphp?Species=Cupressus%20sempervirens, 2019 and (56)]. A homologous allergen, Cry j 7, with similar fruit cross-reactivities was then described in Japanese cedar pollen (*C. japonica*). The study of a cohort of young Japanese patients allergic to Japanese cedar pollen and to fruit showed that 46% are sensitized to Cry j 7 (22). Also, Jun a 7, was also characterized in mountain cedar pollen (*J. ashei*) (49, 57), but no data are available on Jun a 7-dependent PFAS in mountain cedar allergic patients living in area where the trees are growing, mainly in south of USA. We can expect that other trees from the Cupressaceae family such as the Japanese cypress (*Chamaecyparis obtusa*) or the bald cypress (*Taxodium distichum*) also express a pollen allergenic GRP. Purified native Cup s 7 or recombinant Cry j 7 was shown to activate basophils of Cupressaceae allergic patients strongly suggesting a clinical relevance of pollen GRPs (22, 51, 55).

Finally, another GRP allergen was described in 2022 from bell pepper (*Capsicum annuum*), Cap a 7, by studying a Japanese patient allergic to several GRPs, from bell/chili pepper (Cap a 7), peach (Pru p 7), orange (Cit s 7), and from Japanese cedar pollen (Cry j 7). The allergen is present in pulp and skin and in soft and hot *C. annuum* and in *Capsicum chinense*. Like Cry j 7 and Pru p 7, Cap a 7 is able to activate patient’s basophils suggesting a clinical relevance (55).

## Are GRPs panallergens?

Panallergens are widely distributed proteins eliciting IgE reactivity and expressed in different sources with low structural variations in amino-acid sequence and/or structure allowing IgE cross-reactivity. For instance, profilins found in 143 plant species, polcalcins in 51, or LTPs in 92 are considered panallergens (58) (www.allergen.org and www.allergome.org). GRPs fulfill the criteria of wide distribution of the protein; the low structural variations but not the allergenicity in the plant where the proteins are reported since only nine plant species were reported to contain allergenic GRPs up to now (Table 1). We think that, for the moment, the denomination panallergen cannot be assigned to GRPs. Three related issues may be mentioned in that respect: IgE cross-reactivities, conformational epitopes, and requirement for cofactor regulating allergenicity.

**Table 1:** Characteristics of nine GRP allergens and one non allergenic GRP (potato snakin-1^a^).

Protein	English name	Latin name	Family	Exposure	Accession number
Cup s 7	Common cypress	*Cupressus sempervirens*	Cupressaceae	Pollen	LC511610
Jun a 7	Mountain cedar	*Juniperus ashei*	Cupressaceae	Pollen	C0HLQ0
Cry j 7	Japanese cedar	*Cryptomeria japonica*	Cupressaceae	Pollen	CJC05531_1
Pru p 7	Peach	*Prunus persica*	Rosaceae	Food	P86888
Pru m 7	Japanese apricot	*Prunus mume*	Rosaceae	Food	XP_016649029.1
Pru av 7	Sweet cherry	*Prunus avium*	Rosaceae	Food	A0A6P5SVH6
Cit s 7	Sweet orange	*Citrus sinensis*	Rutaceae	Food	A0A067D4T6
Pun g 7	Pomegranate	*Punica granatum*	Lythraceae	Food	A0A218X6T8
Cap a 7	Bell pepper	*Capsicum annuum*	Solanaceae	Food	A0A2G2ZRH2
Snakin-1^a^	Potato	*Solanum tuberosum*	Solanaceae	Food	Q948Z4

^a^ Not reported as allergen.

Other accession numbers for Cup s 7: C0HLL6 (56) and C0HLQ2 (57), and for Cry j 7: C0HLQ1 (57).

GRP, gibberellin-regulated protein.

### Involvement of cross-reactivities

Fruit GRP sensitization is very often associated to Cupressaceae pollen sensitization, which was observed for common cypress in Europe (37, 52) and for Japanese cedar in Japan (22). The explanation of this association is not known as yet, and the primary sensitizer cannot be attributed with certainty to pollen grains or to fruit for the moment. Cross-reactivities might be an explanation of this associated sensitization because percent sequence identities within Cupressaceae are about 90% and 60% with fruits and vegetables. Among plant food, more than 80% sequence identities are found. Therefore, all GRPs should theoretically be cross-reactive and indeed this is confirmed experimentally using recombinant GRPs in direct binding assays (21, 51, 52, 55). However, the cross-reactivities observed in direct binding assays are not systematically retrieved in competitive inhibition assays or in basophil activation test. For instance, despite a positive direct IgE binding, the GRP prototype snakin-1 from potato is unable to inhibit the binding between Cup s 7 and anti-Cup s 7 IgE while Pru p 7 is an efficient inhibitor, and snakin-1 is not able to activate basophils from Cup s 7/Pru p 7 or Cry j 7/Pru p 7 sensitized patients in agreement with the tolerance or partial tolerance of potato consumption by these patients (21, 50, 51, 55). Differences in antibody affinity probably play a role.

**Figure 4:**
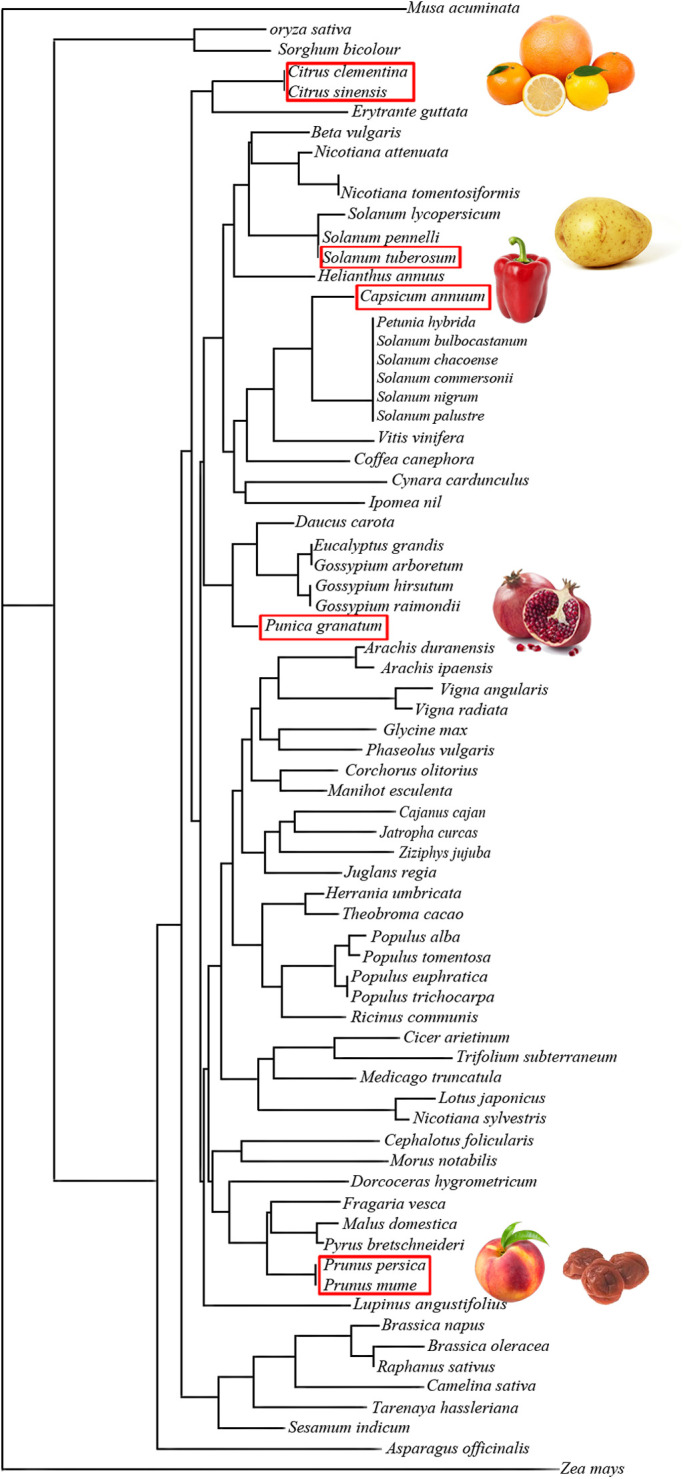
Unrooted tree built up from the multiple alignment of plant food GRPs using the neighboring joining method, showing the phylogenetic relationships among the GRPs of different plant families. GRPs with similar amino-acid sequences are grouped in closely related clusters. Plant food with red frame contains studied GRPs.

Another key issue is the fact that already sequenced GRPs are present in many plants and a BLAST search revealed 250 proteins with 100% sequence identity with the core sequence RCLKYCGICCEK as query. Some of them have been published in a table in (59). Furthermore, 2,450 GASA-related sequences are reported (http://pfam.xfam.org/family/ PF02704#tabview=tab7) in 148 species in the PFAM data bank. However, up to now, GRPs from only nine allergenic sources have been described as allergens: five from fruits, one from vegetable, and three from pollen trees, all belonging to the Cupressaceae family (Table 1). In consequence, despite the ubiquitous nature of GRP and the degree of structural similarities, cross-reactivity is not systematically observed. This may rely on the discrete changes in amino-acid sequences displayed in the different plant families. These changes are located at both extremities of the polypeptide chains, and because of the rigid folding of the molecule, a single point mutation may result in a drastic change of the epitopic regions. According to the amino-acid sequence similarities, 72 GRPs cluster in distinct but rather closely related groups of proteins in the unrooted phylogenetic tree built from their amino-acid sequence alignment, as shown in Figure 4.

IgE reactivity without symptoms was recently exemplified in the case of apple GRP. The apple GRP was studied because apple is a prevalent fruit allergenic source (60). Out of 37 patients mono sensitized to the apple GRP and clinically documented, only one (3%) was allergic to apple with, in addition to the IgE reactivity against apple GRP, specific IgE directed against Mal d 3 (apple LTP) and 29 (78%) had no symptom upon consumption of apple. Therefore, the authors could not conclude on the clinical relevance of apple GRP. It is well known that sensitization (IgE binding) is not always synonymous of allergy. This is reminiscent of what is said about plant cross-reactive carbohydrate determinants although the question is still debated (61).

In summary, GRPs are mainly cross-reactive but not systematically and sensitization may not be clinically relevant. To confer clinical relevance, a cofactor might be required (see below).

### Epitope prediction

**Figure 5:**
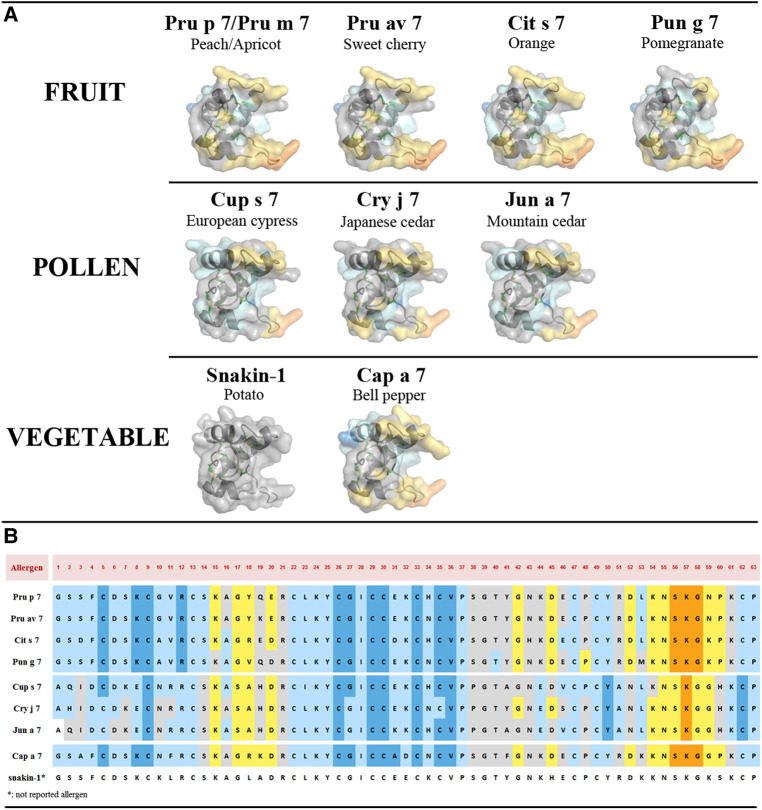
(**A**) Three-dimensional structure modeling of allergenic GRPs inferred by homology modeling using snakin-1 (5E5Q from PDB) as a template with SWISS-MODEL. At least three conformational epitopic regions are predicted using the software DiscoTope 2.0. They are colored yellow and orange. (**B**) GRP allergen sequences with predicted AA involved in epitopic regions. The color codes correspond to DiscoTope propensity scores (62). The highest the score is the highest the propensity to be an epitope is. In ascending order: blue (−20 or less), light blue (−20 to −15), gray (−15 to −12.5), yellow (−12.5 to −5), and orange (greater than −5).

Three-dimensional models based on the snakin-1 structure revealed very similar conformations of the nine allergenic GRPs as expected given the similarities in amino-acid sequences (Figure 5A). A cleft, more or less open, that might accommodate a ligand (see discussion below) can be observed in the different molecules with Pun g 7 being the most open one. Running the software DiscoTope 2.0 can predict at least three epitopic regions. These candidate epitopic regions are predicted based on the 3D structure, thus the predicted results differ even if the amino-acid sequence is conserved (Figure 5B). While those predicted in the not folded loop in the C-terminal region (around K57, lower right part of the molecules in 3D models) are found in all molecules, those on top of the molecules (around AA positions K15 to E20/D20) are not systematically conserved. In this respect, again, Pun g 7 seems different from other fruit GRPs and pollen.

### Ligand or cofactor or both

Another hypothesis to explain the absence of symptoms despite a genuine sensitization might be the absolute requirement of a cofactor to confer a clinical relevance to GRP sensitization and regulate allergenicity of GRPs. Two types of cofactors/regulators can be distinguished: those affecting systemically the host metabolism and subsequently favoring the allergic response and those intrinsically and structurally affecting the molecule by up- or down-regulating its allergenicity.

The first type corresponds to physical exercise, NSAID, alcohol, proton pump inhibitor, stress, concomitant infections, menstruation, or other unknown cofactors. As mentioned above, some of these cofactors were shown to play a role in case of GRP sensitization (32, 38–43). The second type corresponds to the impact, on the allergenicity, of the binding of a specific ligand to the allergen. These interactions were mainly studied with the allergens LTPs and PR10. Lipid binding to LTP can result either in up- or down-regulation of allergenicity by acting on the conformation of the allergen and subsequently on the digestibility and thermostability (63). Also for the PR10 allergen, the allergenicity is differentially affected depending on the ligand, increased with E1-phytoprostane (64), decreased with iron (65), or no effect with quercetin (66).

Besides the small molecule ligands, interactions between macromolecules allergens, while less studied, have also been reported. For instance, Alt a 1, the major allergen from the mold *Alternaria*, and Act d 2, the thaumatin-like protein from kiwi, are able to bind together. These physicochemical results might explain what was clinically reported, i.e., in patients sensitized to Act d 2, 85% are cosensitized to *Alternaria* (67, 68). Also, it was reported that the prevalence of sensitization to Ole e 12, the isoflavone reductase from olive pollen, is 4%–10% in patients with olive pollinosis and rises to 33% when the olive pollen allergic patients are also sensitized to peach (69). This means that interactive sensitizations and challenges might occur. Analyzing the interactions between proteins may therefore bring some new information to understand conditional sensitizations that results in pollen food allergy syndrome. In the case of cypress pollen and peach, at least GRPs are involved, but polygalacturonase or thaumatin-like proteins would deserve to be studied.

**Figure 6:**
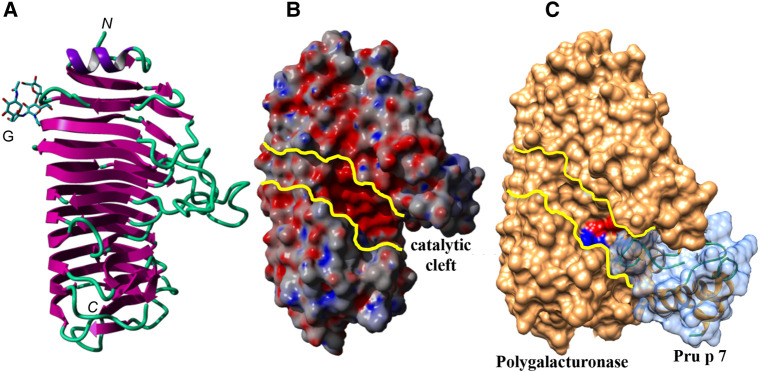
(**A**) Ribbon diagram of the endopolygalacturonase of *F. moniliforme*. The *N*-glycan chain linked to the β-prism backbone is indicated. *N* and *C* indicate the *N-*terminal and *C*-terminal ends of the polypeptide chain, respectively. (**B**) Molecular surface of the endopolygalacturonase showing the catalytic cleft, delineated by yellow lines. (**C**) Docking of Pru p 7 (colored light blue) to the endopolygalacturonase of *F. moniliforme* (colored orange). The amino-acid residues involved in the catalytic cleavage of polygalacturonase chains, are colored red (D191, D212, D213) and blue (R267, K269), respectively. Docking experiments of the modeled Pru p 7 to the endopolygalacturonase of *F. moniliforme* (PDB code 1HG8) (73) used as a target, were performed with GRAMM_X (74, 75) and displayed with Chimera.

With regard to protein–protein interaction including GRP, some data are available for snakin-1 since its exact structure has been resolved by crystallography studies. From the protein–protein interaction database STRING (https://string-db.org/network/ 4113.PGSC0003 DMT400055426), eight proteins were suggested to be able to interact with the potato snakin-1, a defensin, a subtilisin-like protease, an osmotin-like protein, an uncharacterized protein GASA4, a glycolate/glycerate translocator, a ribosomal protein L27, an iron transporter, and a dihydroorotase. Interestingly, the three first proteins were also shown to be allergens: defensin in mugwort and ragweed pollen, peanut, soybean, bell pepper and celery (70); subtilisin-like protease in mold, melon, and Japanese cedar pollen (71); and osmotin (thaumatin-like protein, PR5 protein) in bell pepper, tobacco, and nettle pollen (72). Experimental approaches of such putative ligand binding are, however, missing for the moment with regard to allergenic GRP, but the results obtained with snakin-1 can give some clues to understand potential molecular complexes involved in conditional sensitization, i.e., the sensitization to an allergen which is potentialized by an allergen from a different allergenic source. Whether ligand binding would impact the allergenicity of GRP allergens is still to be explored. In case of an impact on allergenicity, mechanisms might be either the modulation of affinity of the specific IgE to the allergen or the generation of molecular complexes resulting in a “super allergen” able to increase mediator release from the basophils or mast cells. *In silico* docking experiments suggested that Pru p 7 is indeed able to recognize an electronegatively charged patch located at the surface of the polygalacturonases either from the mold Ascomycete *Fusarium moniliforme* (Figure 6) or even from the cypress pollen corresponding to the group 2 allergens (Cup s 2 for *C. sempervirens* for instance). Interestingly, the anchorage of Pru p 7 on the polygalacturonase partially covers the catalytic cleft, thus hampering the accessibility of the potential substrates, e.g. the host Polygalacturonase, to the catalytic residues of the enzyme. Polygalacturonases are expressed in plants and have a role to soften and sweeten fruits during the ripening process. Gibberellin treatment is a strategy to slow down the ripening of the fruits, and the gibberellin-induced excess of GRP might be involved in this process by inhibiting the enzymatic activity of polygalacturonases. These enzymes are allergens described in many plant foods and also in pollen and were reported, for instance, to be at the molecular basis of the cross-reactivity between Cupressaceae pollen and tomato (59). However, such *in silico* speculative docking calculations require experimental validation especially with GRP able to bind to many proteins displaying a protruding electronegative patch.

## Conclusion

In conclusion, many questions on GRPs remain with no response for the moment. Why are GRPs sensitizing allergens in only a few plant foods and only Cupressaceae pollen although they are ubiquitous? One trivial hypothesis could be that the natural *in vivo* extraction of allergens is more or less easily performed from the food or pollen matrix when ingested or inhaled and that the immune system is subsequently differentially exposed. In consequence, for the moment, we cannot say that GRPs are panallergens. The mechanism of the association between food and Cupressaceae pollen GRP sensitization remains also to be understood. Another question is the impact of an exogenous gibberellin treatment used essentially in spray in modern agriculture on the GRP content of plant food and neighboring trees. However, the information of such a treatment is often missing. Also, gibberellin is expressed in the plant in response to a stress but no data are available up to now on the effect of gaseous or particulate stressful pollution on the content of endogenous gibberellin and subsequently GRPs. Whatever is the origin of the gibberellin phythormone increase, exogenous and/or endogenous, this would result in higher numbers or levels of allergenic GRP in the future. GRPs are involved in PFAS and this clinical expression of allergy is now reported to follow a rising trend (76, 77). Obviously more experimental and observational studies are needed to solve these crucial questions.
